# Some Chronic Joint Lesions Following Injury.—II

**Published:** 1909-01-30

**Authors:** 


### SOME CHRONIC JOINT LESIONS FOLLOWING INJURY.—II.

In a previous article we discussed the after effects of injuries of the knee, and pointed out the extreme liability for a condition of permanent weakness or disability to ensue. The shoulder is another joint in which this eventuality is of frequent occurrence. In either case the effect is to render the joint more or less unfit to fulfil the functions for which it is naturally adapted. The functions of the knee are to allow of flexion for the purposes of progression and to form a firm column of support for the body when the upright position is assumed. The "wobbly knee," which has already been described, is unable to do this. The shoulder, on the other hand, is constructed with a view to allowing the greatest possible range of movement in each direction. For this reason we find here a large rounded articular head to the humerus, fitted into a shallow cup, the glenoid fossa. The ligaments which hold it in position are not powerful, and are loosely arranged. In fact, the shoulder-joint receives much more support from the muscles which surround it than it does from the ligaments. Injuries to this joint are more often succeeded by stiffness than they are by a weakening of the joint; and stiffness necessarily interferes with its functions.

In spite of the more or less superficial position of the articulation, and of the fact that it is surrounded by subcutaneous, bony landmarks, it is often a matter of considerable difficulty to diagnose exactly the nature of an injury to this situation. Of course if any of the bones which enter into the formation of the joint are broken, the case should be a comparatively simple one, especially if one has the opportunity of taking a skiagram. But in any case the deformity produced, and the irregularity of the normal outline of the bone, if it can be felt under the skin, should be sufficient to establish the diagnosis.

It is essential to make a thorough examination of the bony points in all cases in which an injury to the shoulder is complained of. Thus the clavicle should be palpated in all its length, and so should the spine of the scapula right up to the tip of the acromion. Next the acromio-clavicular articulation should be examined and compared with that on the sound side. If the joint has been damaged the acromial end of the clavicle will be found to be situated above the level of the acromion itself, or *vice versa*. After this, fracture of the upper end of the humerus must be looked for. The commonest fracture at the upper end of the humerus is that which occurs at the surgical neck. Fracture of the anatomical neck is rarer. There is one simple method of distinguishing between the two. If the index finger of one hand is placed upon the greater tuberosity, while the shaft of the humerus is rotated with the other, the tuberosity will move with the humerus in fracture of the anatomical neck, but will not move if the injury is situated at the surgical neck. Finally the presence of a dislocation must be excluded.

In this article we are mainly concerned, however, with slighter injuries of the joint, uncomplicated by any fracture in the neighbourhood. These are quite common in working men, and are constantly seen in the out-patient department of a general hospital. They are most often the result of a single definite injury, or they may be caused by continued laborious work, such as the ordinary navvy is called upon to do. In the first place the real injury is "a sprain"; that is to say, there is generally some damage to or tearing of the periarticular structures, which causes an effusion of blood into the tissues. There may or may not be a concomitant synovitis. Moderate degrees of effusion into the synovial membrane of the knee-joint are not easily recognised; the swelling along the bicipital groove and the fulness under the deltoid, which are mentioned in text-books of surgery as characteristic of the condition, are not often seen. In fact, examination of the patient in this stage usually reveals no gross abnormality except tenderness and pain when the joint is passively moved.

In the first few days the application of an anodyne lotion, *e.g.* fomentations of lotio plumbi cum opio, is the best treatment, keeping the arm in a sling. But as soon as ever the bruising and swelling begin to subside passive movements should be begun. This should never be delayed beyond a few days. To wait longer than this is to incur a grave risk of allowing adhesions to form.

Any joint, even an absolutely normal one, will develop adhesions in it if kept completely at rest for a considerable time. Adhesions, for instance, may form in the knee-joint after fracture of the femur if passive movement is not judiciously employed. But this will occur far more readily in a joint which is already in a state of inflammation, and in the shoulder-joint particularly. If passive movements are patiently persisted in, no loss of function should ensue, and a good result should be obtained.

Occasionally, however, one sees these cases at some length of time after the accident, and the patient complains that he can hardly move his shoulder at all. Here much improvement may be brought about by moving the joint under anæsthesia. But there are certain things to remember. It is not good practice to attempt this under gas. Complete relaxation must be obtained by deep ether or chloroform anæsthesia. Again, if the adhesions are of long standing, the bone is probably brittle, and any sudden wrenching may fracture it. Further, it is not sufficient to give an anæsthetic once only for this purpose. It is better to do a little at a time, and do it often. One patient, under the writer's care, was given chloroform five times in a fortnight, and each time the range of movement obtained was a little better than before, and the final result was quite satisfactory. In between the movements under anæsthesia, passive movement must be performed daily; and this is the part of the treatment to which the patient objects, because it is exceedingly painful. One reservation must be made. If, as often happens after an injury to the shoulder, osteo-arthritis supervenes, more harm than good will be done by moving the joint under an anæsthetic. The prognosis in this case is exceedingly unsatisfactory.

